# Post-tussive Periorbital Ecchymosis in a Child Without Trauma: A Rare Neuro-Ophthalmic Presentation

**DOI:** 10.7759/cureus.91461

**Published:** 2025-09-02

**Authors:** Ashwin Sridhar, Visvanathan Krishnaswamy

**Affiliations:** 1 Neurological Surgery, Sri Ramachandra Institute of Higher Education and Research, Chennai, IND

**Keywords:** non-traumatic, periorbital ecchymosis, post-tussive, raccoon eye, subconjunctival hemorrhage

## Abstract

Periorbital ecchymosis without preceding trauma is unusual and may be observed after events that increase venous pressure, such as coughing.

We report a case of a three-year-old male who developed bilateral periorbital ecchymosis and subconjunctival hemorrhage following a bout of severe coughing. There was no history of trauma, hematologic disorder, or neoplastic process. Ocular motility and fundus findings were normal. Neuroimaging and systemic investigations were unremarkable. Symptoms resolved spontaneously with conservative management.

Post-tussive periorbital ecchymosis is a rare but benign clinical entity in the pediatric population. Recognition of this presentation is important to avoid unnecessary investigations and to distinguish it from traumatic or systemic causes of periorbital bruising.

## Introduction

Periorbital ecchymosis, colloquially referred to as “raccoon eyes,” is typically associated with a history of trauma with associated pathologies such as anterior cranial fossa fractures or basal skull injuries [1]. Its occurrence in the absence of trauma raises concern of a possible underlying systemic or neoplastic condition, including hematologic disorders, neuroblastoma, and orbital tumors [2,3]. In pediatric patients, these presentations warrant careful evaluation to also exclude abuse or malignancy.

In rare instances, periorbital ecchymosis may result from a sudden increase in venous pressure due to Valsalva maneuvers, such as coughing, vomiting, or sneezing, which can lead to rupture of fragile periorbital capillaries [4,5]. This phenomenon has been reported in isolated case reports involving pertussis infection [6], powerlifting, and vigorous sneezing, but remains exceedingly uncommon in children.

Here, we present a unique case of bilateral periorbital ecchymosis and subconjunctival hemorrhage in a three-year-old child following a bout of severe coughing, without any history of trauma or systemic illness. This report aims to highlight the benign nature of post-tussive ecchymosis and underscore the importance of recognizing this rare presentation to avoid unnecessary investigations.

## Case presentation

A three-year-old male child presented to the emergency room (ER), accompanied by his mother, with swelling and redness of both eyes, which developed suddenly following a bout of coughing. The child, a known asthmatic, had a history of a dry cough for three days and an acute exacerbation of his asthma. There was a worsening of coughing since the morning of his presentation. Approximately four hours prior to the time of arrival at the ER, the child had a bout of severe dry non-productive cough, following which he developed bilateral subconjunctival hemorrhage and periorbital ecchymosis for which the child was brought to the emergency room. The child had no history of fever or rhinitis. The cough, as per the mother, was dry in nature, without any expectoration, and was not associated with any whooping qualities. The child had received all four scheduled pertussis vaccinations (diphtheria, pertussis, and tetanus).

**Figure 1:**
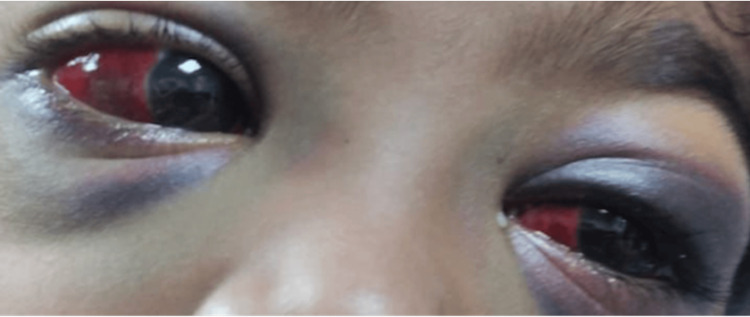
Bilateral periorbital ecchymosis. Image showing bilateral periorbital ecchymosis with subconjunctival hemorrhage on presentation in the emergency department.

On examination, the child was alert and systemically well. Ocular evaluation revealed bilateral periorbital ecchymosis and subconjunctival hemorrhage (Figure 1). Pupillary responses, extraocular movements, and fundus examination were all normal. There was no orbital bruit. Neurologic and systemic examinations were unremarkable. On examination, the cough was dry in nature, without any expectoration, and was not associated with any whooping quality. The pediatrician was consulted, and the opinion was that the cough was probably an acute exacerbation of asthma. The patient's cough was treated using anti-tussive agents, and asthma was managed using nebulization and bronchodilators.

Given the absence of trauma or systemic symptoms that would cause the periorbital ecchymosis, a targeted diagnostic approach was undertaken. Laboratory investigations, including complete blood count, coagulation profile, and peripheral smear, were within normal limits. No polymerase chain reaction test was undertaken, as the patient's history and presenting symptoms did not point toward pertussis infection. A chest X-ray and non-contrast CT (NCCT) scan of the brain and orbits showed no evidence of skull fracture, intracranial hemorrhage, or orbital mass. A whole-body PET scan excluded neoplastic pathology, including neuroblastoma.

**Figure 2:**
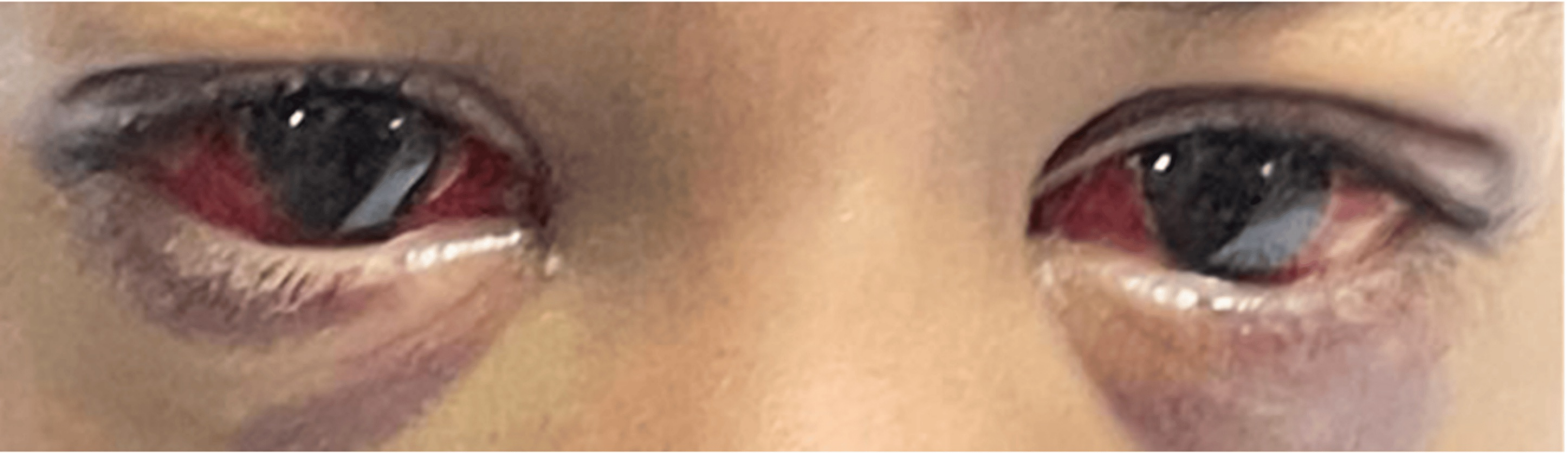
Resolving periorbital ecchymosis. Image showing resolving bilateral periorbital ecchymosis with subconjunctival hemorrhage on presentation in the outpatient clinic after two weeks.

The patient was managed conservatively with observation. The periorbital ecchymosis and subconjunctival hemorrhage resolved spontaneously over the next two weeks without intervention (Figure 2).

## Discussion

Periorbital ecchymosis is a well-recognized sign in traumatic brain injuries, particularly those with associated anterior cranial fossa fractures. In contrast, spontaneous ecchymosis without trauma is rare and often prompts extensive evaluation to rule out serious underlying pathology. In pediatric patients, differential diagnoses include hematologic disorders (e.g., thrombocytopenia), orbital tumors (e.g., rhabdomyosarcoma), and systemic malignancies such as neuroblastoma [2,3].

The current case is notable for its benign etiology, i.e., post-tussive, following an acute asthma exacerbation. Similar cases have been reported in the literature, including bilateral eyelid ecchymosis associated with pertussis-induced coughing paroxysms [6], vomiting-induced bruising [4], and sneezing-related raccoon eyes. However, most of these reports involve older children or adults, making this case particularly rare in a three-year-old patient.

The pathophysiology is presumed to involve transient elevation of intrathoracic and venous pressure during coughing, leading to rupture of delicate periorbital capillaries. This mechanism is consistent across reported cases involving Valsalva-like maneuvers [5]. Unlike traumatic or neoplastic causes, post-tussive ecchymosis is self-limiting and resolves spontaneously, as observed in our patient.

What distinguishes this case is the absence of systemic symptoms, normal imaging and hematologic workup, and rapid resolution with conservative management. The child’s history of asthma may have predisposed him to forceful coughing, amplifying venous pressure and triggering the ecchymosis. Importantly, the patient had received all scheduled pertussis vaccinations, reducing the likelihood of pertussis-related complications.

This case reinforces the need for clinicians to consider benign etiologies in the differential diagnosis of raccoon eyes, especially when trauma and systemic disease are excluded. A thorough history and focused investigations can prevent unnecessary imaging and invasive testing, which is particularly crucial in pediatric care.

## Conclusions

This case highlights a rare but benign cause of bilateral periorbital ecchymosis and subconjunctival hemorrhage in a child following severe coughing. Post-tussive ecchymosis should be included in the differential diagnosis of raccoon eyes in the absence of trauma or systemic disease. Awareness of this entity can prevent misdiagnosis and unwarranted investigations.
